# Identification and quantification of plasma free salusin-β, an endogenous parasympathomimetic peptide

**DOI:** 10.1038/s41598-017-08288-0

**Published:** 2017-08-15

**Authors:** Kazumi Fujimoto, Akinori Hayashi, Yoshio Kodera, Tatsuya Saito, Takuya Toki, Akifumi Ogawa, Yuji Kamata, Koji Takano, Hideki Katakami, Masayoshi Shichiri

**Affiliations:** 1 Department of Endocrinology, Diabetes and Metabolism, Kitasato University School of Medicine, 1-15-1 Kitasato, Minami-ku, Sagamihara, Kanagawa 252-0374 Japan; 2 Department of Physics, Laboratory of Biophysics, Kitasato University School of Science, 1-15-1 Kitasato, Minami-ku, Sagamihara, Kanagawa 252-0373 Japan; 3 Center for Disease Proteomics, Kitasato University School of Science, 1-15-1 Kitasato, Minami-ku, Sagamihara, Kanagawa 252-0373 Japan; 4 Teikyo University Chiba Medical Center, 3426-3 Anesaki, Ichihara, Chiba 299-0111 Japan

## Abstract

Salusin-β is an endogenous parasympathomimetic proatherosclerotic peptide. Salusin-β was initially predicted from bioinformatic analyses and later immunologically detected in human biofluids. However, elucidation of salusin-β bioactivity has faced enormous challenges because of its unique physicochemical characteristics that cause it to strongly adhere to laboratory apparatus materials. In the strictest sense, the discovery of bioactive peptides is not complete until their exact native sequences have been confirmed in the peripheral circulation. In this study, we determined the plasma molecular form and levels of free salusin-β to determine its pathophysiological significance. Ultra-high-yield enrichment and preseparation of non-tryptic human plasma was followed by LC-MS/MS, and full-length salusin-β and seven different endogenous fragment sequences were identified. We established a new ELISA that specifically detects plasma free salusin-β without cross-reacting with any of its identified endogenous fragments. Free salusin-β levels exhibited a profound early morning nadir and rapidly decreased in response to parasympathetic nervous augmentation. Our technical advance in plasma native peptide analysis successfully identified a hard-to-detect bioactive peptide, salusin-β, together with its formerly unrecognized fragments, and further suggests that conventional immunological measurements of target peptides may not be fully representative.

## Introduction

Salusin-β is a potent bioactive peptide that we originally predicted using *in silico* analysis of a human cDNA library^1^. Salusin-β-like immunoreactivity was later demonstrated in human plasma and urine^2–4^. Salusin-β exerts combined systemic and local biological actions, including hypotensive and bradycardic effects^1^, that are mediated via systemic parasympathetic stimulation and negative cardiotropic inotropism^5^. Central salusin-β regulates hemodynamic homeostasis by inducing antidipsogenesis^6^, blood pressure elevation^7^ and stimulation of vasopressin and oxytocin secretion^1, 8^. Peripheral activities of salusin-β include its potent proatherosclerotic effects^9–12^ and suppression of cardiac remodeling^13^. In addition to such overwhelming biological activities, it has also been proposed as a biomarker for certain human diseases^12, 14^.

Despite its potent and unique activities, measurement of human plasma salusin-β concentrations using commercially available kits described in the literature is largely inconsistent^15–20^. Highly purified synthetic salusin-β, immediately after reconstitution with distilled water, tightly adheres to various plastic and glass consumables in medical and laboratory use^14, 21–23^. Such unusual physicochemical properties of salusin-β can be circumvented by the addition of a low concentration of non-ionic detergent^2, 21^. The processing of salusin-β and its precursors remains largely unknown. Although full-length salusin-β appears to bind to plasma proteins, its binding capacities and affinities are as yet unknown^14^ and such binding is likely to confound the published serum salusin-β measurements.

We are in the process of successfully identifying plasma native peptides using LC-MS/MS^24, 25^, and unveiling a variety of fragments and precursors of many classical bioactive/biomarker peptides in the human peripheral circulation. Among the many circulating proteins we have already identified, a significant number have amino acid substitutions, amino acid modifications^26^, or both. These changes may account for the altered antibody binding capacities. Measurements of plasma or serum peptide hormone levels almost always utilize antibodies recognizing partial sequences with high antigenicity. However, if cleaved peptides or precursor proteins are also present in human plasma, conventional assays employing such antibodies may not determine the exact peptide concentrations. Some commercially available ELISA kits exert unexpected reactivity to known or unknown antigens and it is recommended that results be interpreted with extreme caution^27–31^.

Characterizing the exact amino acid sequences of plasma bioactive and biomarker peptides, together with their endogenous fragments or precursors, remains an important unmet challenge in the fields of analytical biochemistry and health sciences. This is because of unresolved technical difficulties that prevent circumvention of known and unknown confounding factors, such as the myriads of cocirculating high abundance proteins. Here, we use LC-MS/MS analysis of non-tryptic human plasma to identify the exact amino acid sequence of salusin-β and examine whether its precursors and derivatives circulate in human peripheral blood. Our results prompted us to measure exact plasma concentrations of full-length salusin-β peptide separately from related native sequences that cocirculate in human plasma.

## Results

### Identification of circulating salusin-β and its fragments

**Figure 1:**
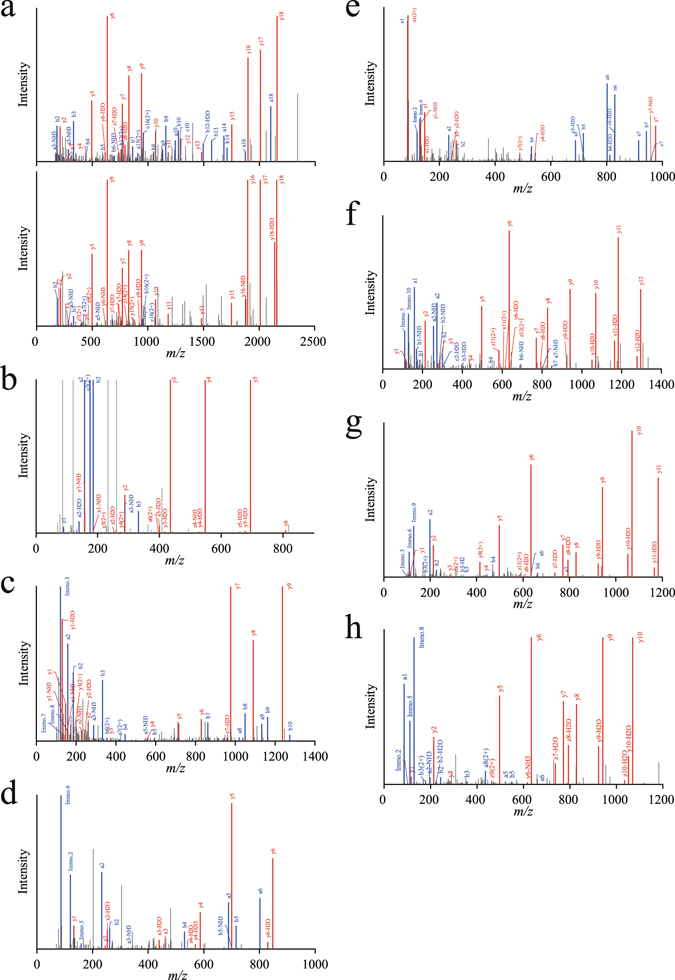
MS/MS spectra for salusin-β and its endogenously fragmented peptides directly identified from healthy human plasma. Human plasma extracted using the improved differential solubilization method was prefractionated and subjected to LC-MS/MS analysis. (**a**) MS/MS spectra with the sequence assignments of fragment ions correspond to salusin-β “AIFIFIRWLLKLGHHGRAPP” with a *m/z* of 586.35 (molecular mass of 2,341.37 Da). The MS/MS spectra of synthetic salusin-β (upper panel) was compared to the original spectrum observed in a plasma sample (lower panel) to confirm the putative identification. (**b–h**) MS/MS spectra corresponding to partial sequences of salusin-β directly identified using non-tryptic human plasma samples are presented: (**b**) salusin-β(1–7), (**c**) salusin-β(1–11), (**d**) salusin-β(4–10), (**e**) salusin-β(4–11), (**f**) salusin-β(8–20), (**g**) salusin-β(9–20), (**h**) salusin-β(10–20). MS/MS spectra were deconvoluted into singly charged ions from the observed spectra and peaks were assigned theoretical *m/z* values for fragment ions. The *m/z* differences between theoretical and observed values for most assigned peaks were less than 0.01 Da. The identified cleaved peptide sequences of salusin-β after PEAKS studio database searches are presented. The annotations of the identified matched amino terminus-containing ions are shown in blue and the carboxyl terminus-containing ions in red.

Pooled plasma was depleted of high-abundance proteins and further enriched for the low molecular weight (MW) native peptides fraction by removing residual plasma high MW proteins. Enriched eluates containing low MW native peptides that were previously unbound or bound to carrier proteins were subjected to pre-separation using RP-HPLC followed by LC-MS/MS. The data obtained were subjected to a PEAKS Studio database search based on *de novo* sequences. This resulted in the identification of the full-length 20 amino acid residue, salusin-β (Fig. 1a) and 7 additional cleavage products (Fig. 1b–h). The mass spectrometry data have been deposited to the ProteomeXchange Consortium via the PRIDE^32^ partner repository (Table 1).

**Table 1:** Salusin-β and its seven endogenous fragments detected in human plasma.

Peptides	Sequence	PSMs^*1^	−10lgP^*2^	*m/z* (observed)	Molecular Charge	Retention time	Molecular weight (calculated)	Mass difference (ppm)	LC-MS Data No.^*3^ (PRIDE)	Scan No.
salusin-β	AIFIFIRWLLKLGHHGRAPP	6	68.1	586.3492	4	80.6	2341.3687	−0.4	MS092	20571
salusin-β(1–7)	AIFIFIR	2	30.9	440.2740	2	61.24	878.5378	−4.8	MS095	16060
salusin-β(1–11)	AIFIFIRWLLK	2	46.4	710.4471	2	79.27	1418.8801	−0.3	MS094	20373
salusin-β(4–10)	IFIRWLL	2	31.7	480.8035	2	76.71	959.5956	−3.3	MS095	19690
salusin-β(4–11)	IFIRWLLK	2	25.5	544.8522	2	74.4	1087.6906	−0.6	MS095	19127
salusin-β(8–20)	WLLKLGHHGRAPP	10	49.1	494.6212	3	29.5	1480.8416	0.1	MS093	7577
salusin-β(9–20)	LLKLGHHGRAPP	4	44.1	432.5946	3	13.07	1294.7622	−0.2	MS093	3155
salusin-β(10–20)	LKLGHHGRAPP	3	38.4	394.8999	3	8.91	1181.6781	−0.3	MS089	2096

^*1^PSMs, Peptide signal matches. ^*2^False discovery ratio thresholds of 1%, 0.1%, 0% estimated with the target-decoy method^45^ are the values of −10lgP of 21.0, 26.9, 31.9, respectively. ^*3^Number of LC-MS raw data deposited in ProteomeEXchange Consortium^32^ with the identifier PXD003533.

### Free salusin-β ELISA

**Figure 2:**
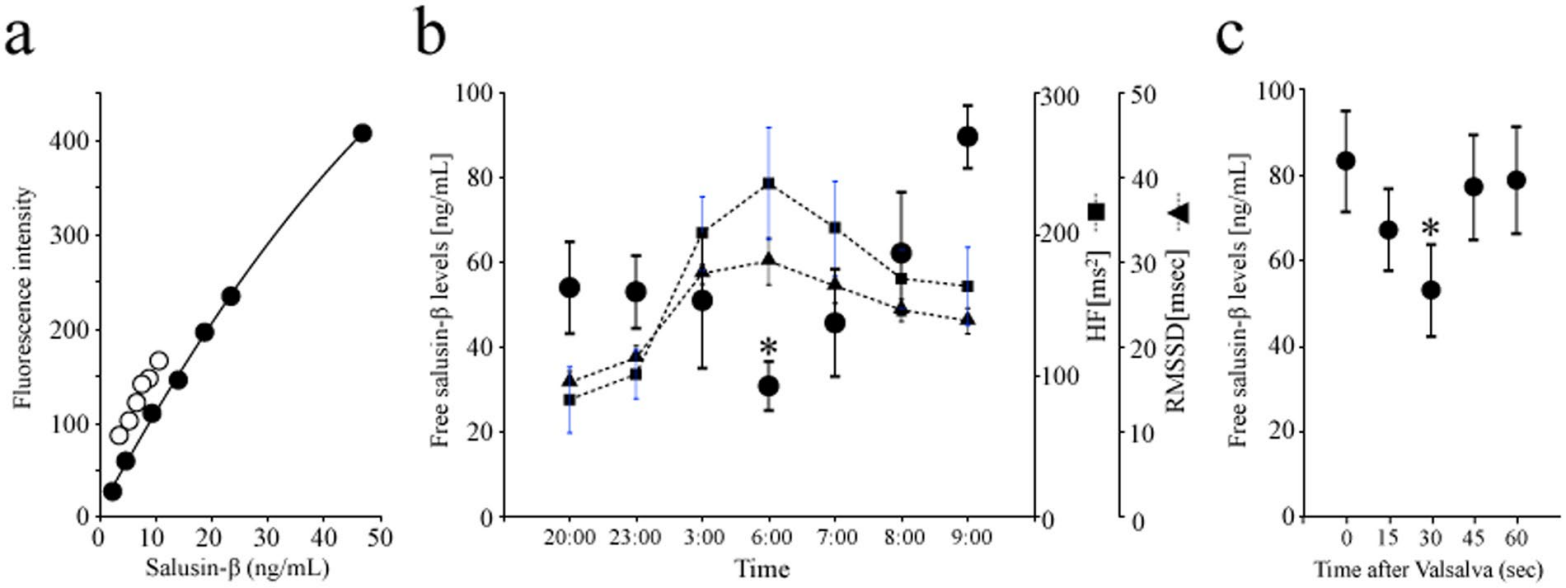
Plasma salusin-β concentrations in healthy volunteers. (**a**) Apparent parallelism between salusin-β standard and human plasma samples by sandwich ELISA. Serially diluted synthesized salusin-β reconstituted and diluted with 0.025% NP40/PBS to concentrations of 2.3–46.8 ng/mL (1.0–20 nmol/L, closed circles) was compared with serially diluted human plasma samples (1:5–1:20, open circles). (**b**) Night-time fluctuations of plasma free salusin-β levels measured in 5 healthy volunteers under ambulatory conditions between 8 pm through to 9 am the next morning, compared against physiological autonomic functions determined by heart rate variability measurements. Closed squares and closed triangles with dotted lines represent changes of parasympathetic nervous function as assessed by high frequency components (HF) and the root mean square of successive differences of normal-to-normal intervals (RMSSD). Each closed point with horizontal bars indicate the mean ± SE levels. *P < 0.05 compared against 9 am samples. (**c**) Effects on plasma salusin-β after Valsalva maneuver. Seven healthy subjects (3 males and 4 females) were given Valsalva maneuver, and plasma salusin-β levels were measured before and 15, 30, 45 and 60 s after the start of the maneuver. *P = 0.0078 compared against baseline values.

A specific sandwich ELISA was established and validated for the direct measurement of plasma free salusin-β. However, we found that addition of NP-40, a non-ionic detergent normally used to prevent salusin-β loss, at concentrations ≥0.05% actually reduced salusin-β binding to its antibody. To overcome this issue, we used 0.025% NP-40 to dissolve high-purity salusin-β and for subsequent experimental uses of salusin-β solution. A standard absorbance curve was generated using synthetic human salusin-β peptide diluted to 2.3–46.8 ng/mL (1.0–20 nmol/L). To evaluate parallelism, serial dilutions of extracted human plasma were assayed, yielding a highly linear relationship with an R^2^ value of 0.99 (*P* < 0.0001; Fig. 2a). The intra-assay CV was 4.8% (n = 7) and the inter-assay CV 17.1% (n = 10). The minimum detection limit was 1.12 ng/well (0.48 nmol/well) and the ED_50_ was 18.5 ng/well (43.3 nmol/well). We synthesized the seven salusin-β fragment sequences we identified in human plasma and found that none had greater than 0.03% cross-reactivity when subjected to our salusin-β ELISA (Table 2).

**Table 2:** Crossreactivites of each endogenous salusin-β fragment identified by LC-MS analysis in a salusin-β sandwich ELISA.

Endogenous fragments	Crossreactivity (%)
Salusin-β(1–7)	0.02
Salusin-β(1–11)	0.02
Salusin-β(4–10)	0.01
Salusin-β(4–11)	0.03
Salusin-β(8–20)	0.02
Salusin-β(9–20)	0.03
Salusin-β(10–20)	0.03

### Pathophysiological implications of plasma free salusin-β

Free salusin-β measured during daytime did not show any appreciable circadian variations. However, overnight blood sampling revealed markedly reduced levels at 6 am (Fig. 2b). This early morning nadir was profound and coincided with night-time parasympathetic augmentation as demonstrated by elevated high frequency components (HF) and the root mean square of successive differences of normal-to-normal intervals (RMSSD) values (Fig. 2b). The magnitude of this decrease in free salusin-β was far more pronounced than that observed previously for total salusin-β^2, 3^.

To examine the direct effect of parasympathetic nervous stimulation, we measured free salusin-β levels before and after the Valsalva maneuver using the same plasma samples that we previously used to determine total salusin-β. Unlike total salusin-β levels, which gradually decreased following the Valsalva maneuver^3^, free salusin-β levels did not show any significant changes at 1, 5, 10, 20, and 30 min following the maneuver. We repeated the experiment to investigate whether the Valsalva maneuver exerted more rapid changes. Plasma free salusin-β levels were reduced within 30 s, and these levels returned to the baseline level after 45 s (Fig. 2c).

We investigated whether plasma free salusin-β is under the influence of physiological stimuli that modulate hemodynamic and body fluid status. Compared with the values obtained after sitting for 15 min (74.2 ± 10.0 ng/mL), plasma salusin-β levels were unaffected after 30 min in the supine position (73.9 ± 8.4 ng/mL) and after 60-min in the standing position (72.4 ± 6.9 ng/mL). Drinking 20 mL/kg body weight of water did not significantly suppress plasma salusin-β after 60 min (67.4 ± 16.8 vs. 61.8 ± 14.4 ng/mL, ns). Hypertonic saline infusion, a powerful stimulus of vasopressin release, also did not significantly affect plasma salusin-β levels after 60 min (42.8 ± 14.9 *vs*. 45.1 ± 17.2 ng/mL, ns). Combined, these results show that stimulation of vasopressin release is unaccompanied by changes in free salusin-β levels.

**Figure 3:**
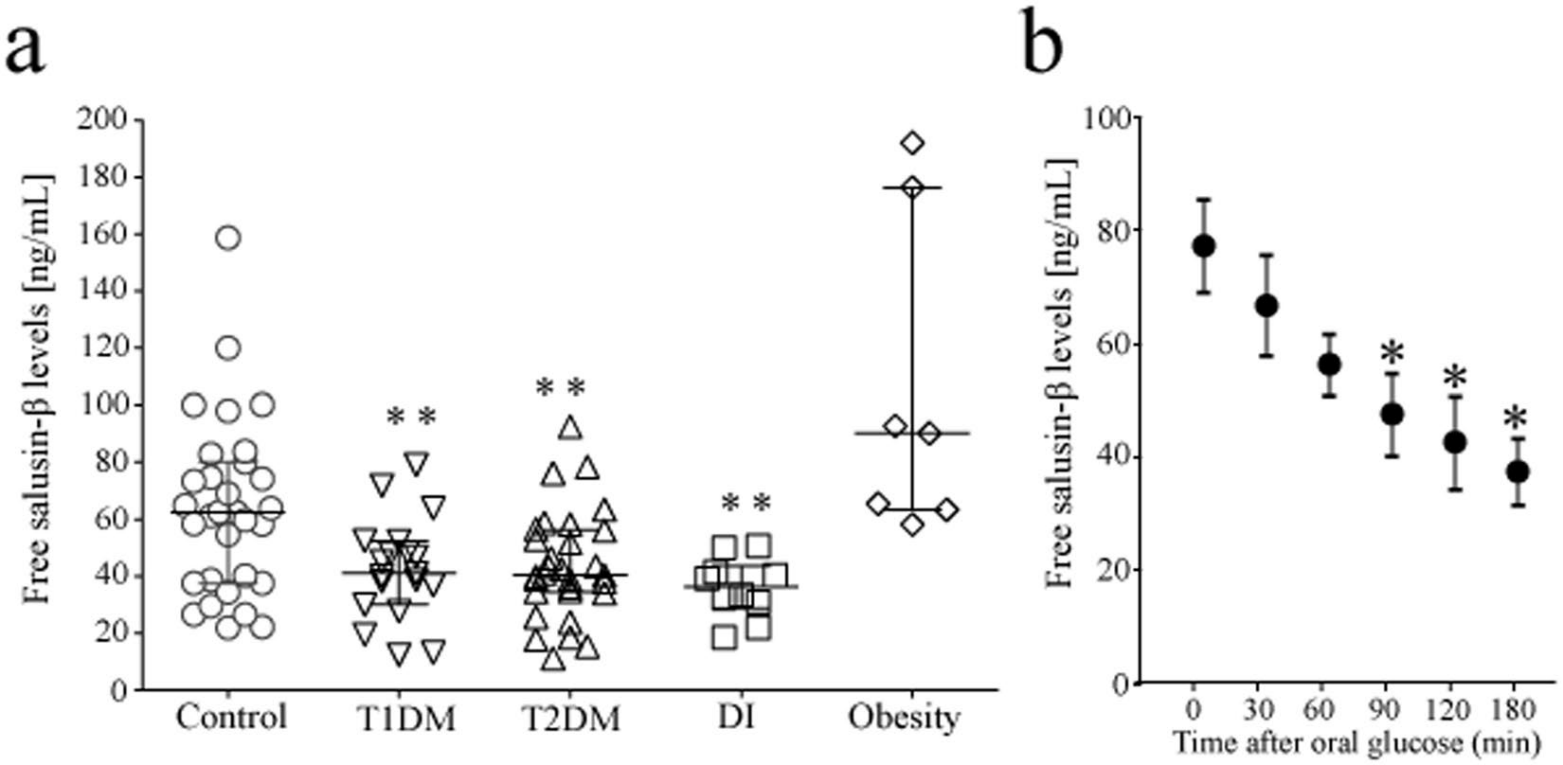
Plasma salusin-β concentrations in healthy non-obese and obese subjects and in patients with diabetes mellitus and diabetes insipidus. (**a**) Plasma free salusin-β levels were determined in 20 patients with type 1 diabetes (T1DM, 11 males and 9 females), 31 patients with type 2 diabetes (T2DM, 15 males and 16 females), and 10 patients with panhypopituitarism and complete central diabetes insipidus (DI, 4 males and 6 females) and in 7 non-diabetic subjects with obesity (4 males and 3 females) and compared with the levels of 28 healthy non-obese subjects (14 males and 14 females). Each point represents the salusin-β level found in a single subject and the horizontal bars indicate the median and whiskers extending between the 25^th^ and 75^th^ percentile values of each group. A one-way ANOVA followed by a Mann-Whitney U post hoc test was used to compare free salusin-β levels for each pair of groups. **P < 0.005, compared against the control group. b. Blood samples were collected from ten non-diabetic subjects before and at 30, 60, 90, 120 and 180 min after oral ingestion of 75 g glucose. Plasma glucose, immunoreactive insulin and free salusin-β levels were measured. Data points with bars represent mean ± SE. *P < 0.05, compared against the baseline value.

To further explore the conditions affecting plasma free salusin-β levels, we measured levels in patients with diabetes and panhypopituitarism, and in non-diabetic obese subjects. Patients with type 1 and type 2 diabetes, but not nondiabetic obese subjects, had significantly reduced plasma free salusin-β levels compared with healthy controls (*P* < 0.005, Fig. 3a). Patients with panhypopituitarism combined with complete central diabetes insipidus also had reduced plasma free salusin-β levels (*P* < 0.005) compared with healthy controls. This finding supports the hypothesis that peripheral salusin-β might partly originate in the pituitary. Multivariate analyses confirmed that type 1 diabetes, type 2 diabetes and panhypopituitarism were independently associated with reduced free salusin-β values (β = −0.551, F = 13.742, P < 0.0005; β = −0.629, F = 17.186, P < 0.0001, β = −0.474, F = 14.876, P < 0.0005, respectively), whereas age, gender and body mass index were not selected as independent variables influencing these levels. We analyzed whether the exact glycemic profiles of our diabetic patients were related to their plasma free salusin-β values. Neither mean glucose values nor glycemic SD data obtained during the 48-h CGM recordings^33^ was significantly correlated with plasma free salusin-β values. HbA1c, glycated albumin, serum LDL cholesterol, and serum HDL cholesterol levels also did not correlate with plasma free salusin-β. In our non-diabetic population, oral intake of 75 g glucose suppressed the free salusin-β levels and the effect lasted for at least 180 min (Fig. 3b).

## Discussion

The plasma proteins that have been characterized to date have widely variable concentration range between highly expressed proteins and low-abundance bioactive/biomarker peptides. This variability represents a major limiting factor for identifying bioactive/biomarker peptides. To overcome the issue, the present study utilized a high-yield plasma extraction technique^24^ to efficiently deplete plasma high abundant proteins and subsequently used mass spectrometry to characterize plasma low MW peptides. Our extraction protocol did not employ the proteolytic digestion that is often used in bottom-up proteomics, and successfully enriched plasma native peptides after releasing those associated with high MW plasma proteins. Consequently, the identified fragment sequences are considered to be native forms that are consistently present in the human peripheral circulation. The technical advance described herein has enabled us to identify seven distinct endogenous cleavage products of salusin-β in addition to full-length salusin-β.

Because endogenous salusin-β fragments share identical sequences with full-length salusin-β, they may modulate the well-described biological activities of salusin-β by competing with cell surface salusin-β receptors. Furthermore, these fragments may interfere with plasma salusin-β measurements because the currently available salusin-β antibodies recognize only the C-terminal sequences. Experiments using synthetic salusin-β and stable isotope labelled salusin-β revealed that salusin-β not only tightly binds to experimental plasticware such as HPLC/mass spectrometry circuits, but also abundantly binds to plasma proteins. Together, these features make quantification using spike-in salusin-β peptide extremely difficult. Moreover, the seven fragments also appeared to have varying degrees of the same physiocochemical features. Consequently, we were unable to establish a quantitative salusin-β assay using mass spectrometry. We raised two antibodies that specifically recognize the C-terminal and N-terminal end 6 amino acid residues of salusin-β and successfully established a sandwich ELISA that directly detected full-length salusin-β without cross-reacting with any of the endogenous fragments identified in the current LC-MS/MS analysis. Because our ELISA does not require extraction of the plasma, it measures levels of circulating salusin-β in the plasma that are unbound to carrier proteins. Therefore, we consider our ELISA to be suitable for directly detecting plasma free salusin-β.

The plasma free salusin-β levels we detected in the current ELISA were clearly higher than those described in the literature^15–20^. Almost all previous reports that determined plasma salusin-β levels used commercial salusin-β kits. These researchers were unaware both of the existence of the endogenous fragments identified in the present study, as well as the unusual physiocochemical characteristics of salusin-β that make it highly adherent to laboratory plasticware^23, 34^. We found that the non-ionic detergent NP-40 successfully prevented salusin-β from sticking to experimental tubes and tips^2, 34^. Detergents must always be used when redissolving salusin-β peptides, and when handling samples containing salusin-β. However, we also found that conventional NP-40 concentrations could potentially impair salusin-β-binding to its antibodies. In light of this finding, our previously reported measurements of plasma total salusin-β levels using a different C-terminal antibody and a high NP-40 concentration may have been inaccurate^2, 3^. In the present study, we found that addition of 0.025% NP-40 successfully circumvented its binding to polypropylene, polystyrene and glass, without affecting its binding to antibodies. Therefore, we employed this condition throughout our experiments and successfully established a direct sandwich ELISA that detects plasma free salusin-β levels without requiring extraction. The concentrations of salusin-β required to modulate systemic hemodynamics^1, 5, 14^ may be lower than the actual free salusin-β levels as demonstrated in the present study.

The present study also supported our previous observation that salusin-β has an early morning nadir^3^ and further showed that free salusin-β exhibits a far more profound decrease in the early morning in comparison with total salusin-β. The time course relationship revealed a simultaneous plasma free salusin-β nadir and a nocturnal parasympathetic augmentation, as demonstrated by remarkable increases in HF and RMSSD. Furthermore, plasma free salusin-β rapidly decreased following a Valsalva maneuver, which is a well-established and safe technique that induces a vagal reflex^35, 36^. In contrast to plasma total salusin-β levels, which reached the 30 min minimum after the maneuver, free salusin-β rapidly decreased and then returned to the baseline level within one min. These data suggests that circulating free salusin-β levels are suppressed following physiological parasympathetic stimulation. Therefore, a negative feedback relationship may exist between the parasympathetic nervous system and free salusin-β to counteract hemodynamic derangements caused by sudden parasympathetic augmentation.

Although salusin-β modulates hemodynamics by stimulating arginine vasopressin release^8, 37–39^, plasma free salusin-β levels were neither affected by hypertonic saline infusion nor postural changes. We found that salusin-β levels were significantly suppressed following endogenous insulin secretion after a 75 g oral glucose tolerance test. Furthermore, patients with both type 1 and type 2 diabetes had significantly reduced free salusin-β levels when compared against non-diabetic controls. All type 1 diabetic patients and the majority of type 2 diabetic patients received insulin therapy. Almost all of the remaining type 2 diabetic patients either received oral hypoglycemic reagents such as sulfonylurea, or glinides which stimulate insulin secretion from pancreatic beta cells. To exclude the possibility that free salusin-β levels in diabetics were affected by glycemic control, we analyzed CGM records of all of the diabetic patients and found no significant effect of mean glucose or glycemic excursion on plasma free salusin-β. These results suggest that free salusin-β levels are suppressed in response to insulin. Patients with panhypopituitarism combined with complete diabetes insipidus showed lower plasma free salusin-β levels, suggesting a significant contribution of neuroendocrine sources to peripheral free salusin-β levels in humans.

In summary, we successfully identified seven native cleavage products of salusin-β in addition to full-length salusin-β in the human peripheral circulation and established a specific ELISA that exclusively detects plasma free salusin-β levels. Free salusin-β levels are sufficient to modulate systemic hemodynamics, and are under regulation of parasympathetic nervous stimulation or insulin secretion. The technical advance described herein paves the way for more accurate assays that specifically measure plasma levels of bioactive peptides. In particular, such assays will allow characterization of endogenous low MW peptide hormones in human plasma that conventional methods have so far been unable to identify.

## Materials and Methods

### Study participants

A total of 28 healthy volunteers (14 men and 14 women, age 46.5 ± 13.2), 51 diabetic patients (type 1 diabetes, 11 men and 9 women, age 54.9 ± 4.0; type 2 diabetes, 15 men and 16 women, age 54.5 ± 4.5), 10 patients with panhypopituitarism with complete diabetes insipidus (4 men and 6 women, age 46.3 ± 4.6), and 7 patients with obesity without diabetes (4 men and 3 women, age 33.3 ± 2.4) provided blood samples. None of the healthy volunteers had any current medical problems. Patients with diabetes mellitus or diabetes insipidus were diagnosed and recruited as previously described^40, 41^. The protocols were approved by the Kitasato University Medical School Ethics Committee and informed consent was obtained from all participants. All study methods were performed in accordance with the relevant guidelines and regulations of Kitasato University Medical School.

### Plasma sample collection

Blood samples from the above subjects were collected into vacutainers containing Na_2_-EDTA (1.5 mg/mL), and plasma was separated immediately in a refrigerated centrifuge and stored in aliquots at −30 °C until processing. For LC-MS/MS analysis, plasma obtained from four healthy volunteers were combined without addition of any detergents and immediately stored at −80 °C.

### Mass spectrometry analysis

Thawed plasma was processed according to the differential solubilization method^24, 42^ with the following minor modifications. A 50-μL plasma sample diluted with 100 µL of 7 M urea, 2 M thiourea, and 20 mM dithiothreitol solution was slowly dropped into 2 mL of ice-cold acetone and immediately stirred at 4 °C for 1 h and centrifuged at 19,000 × *g* for 15 min at 4 °C. The precipitate was then resuspended in 1 mL 80% acetonitrile and 12 mM HCl, mixed at 4 °C for 2 h, centrifuged again, and the supernatant lyophilized and stored at −80 °C. Lyophilized peptides were redissolved in 1 × Invitrosol (Life Technologies) and 100 mM ammonium hydrogen carbonate^25^, and combined to give a total of 480 µL of original plasma to be injected onto a 2.0-i.d. × 100-mm C18 reversed phase (RP) column (Cadenza CD-C18; Imtakt) attached to a HPLC system (Nanospace SI-2; Shiseido Fine Chemicals). Extracted peptides were separated at 1 min intervals and combined into 8 fractions using cyclic sample pooling^43^. All HPLC fractions were then lyophilized and redissolved in 32 µL of 500 ng/mL NV10 (AMR) and 8 µL (120 µL of original plasma) was injected onto a C18 0.075- × 20-mm trap column (Acclaim PepMap 100; Thermo Fisher Scientific) and then eluted onto a C18 0.075- × 120-mm analytical column (Nano HPLC Capillary Column; Nikkyo Technos) configured to an EASY-nLC 1000 HPLC system (Thermo Fisher). The mobile phases consisted of (A) 0.1% formic acid and (B) 0.1% formic acid and 90% acetonitrile, and the flow rate of the mobile phase was set at 300 nL/min. The mobile phase flow was programmed as follows: 8–28% B (0–60 min), 28–50% B (60–70 min), 50–100% B (70–72 min), and 100% B (72–90 min). Separated peptides were subjected to Q-Exactive™ (Thermo Fisher) operated in data-dependent mode to automatically switch between full-scan MS and MS/MS acquisition. Full scan mass spectra (m/z 350–1200) were acquired in the Orbitrap instrument (Thermo Fisher) with 70,000 resolution at m/z 200, after accumulation of ions to a 1 × 10^6^ target value. The ten most intense peaks with charge state more than two from the full scan were selected with an isolation window of 2.4 Da, and fragmented in the higher energy collisional dissociation cell with normalized collision energy of 27%. Tandem mass spectra were acquired in the Orbitrap mass analyzer with a mass resolution of 17,500 at m/z 200, after accumulation of ions to a 5 × 10^4^ target value. The ion selection threshold was 7 × 10^5^ counts, and the maximum allowed ion accumulation times were 120 ms for full MS scans, and 200 ms for tandem mass spectra. Typical mass spectrometric conditions were as follows: spray voltage, 2 kV; no sheath and auxiliary gas flow; heated capillary temperature of 250 °C; and dynamic exclusion time, 30 s.

Database searches were performed using the SwissProt_2015_02.fasta database (selected for Homo sapiens; 20,199 entries), and PEAKS Studio (version 7.0, Bioinformatics Solutions) was used to perform *de novo* peptide-sequence-based database searches from MS and MS/MS spectra of peptides. The search parameters were as follows: enzyme, no enzyme; variable modifications, acetyl (N-term), amidated (C-term), oxidation (M); peptide ion mass tolerance, 6 ppm; fragment ion mass tolerance, 0.02 Da. The false discovery rate was set at 1%. The raw mass spectrometry data have been deposited into the ProteomeEXchange Consortium database^32^ and the information on respective identifiers has been added to Table 1.

### Production and purification of salusin-β antibodies

Synthetic peptide, [Cys^0^]-PEG-GRAPP (Scrum), was pretreated with a protein crosslinking and fixation reagent, mixed with untreated [Cys^0^]-PEG-GRAPP, coupled to maleimide-activated mariculture keyhole limpet hemocyanin (Pierce), and immunized into Japanese white rabbits. This polyclonal C-terminal salusin-β antiserum was purified using a Melongel IgG purification kit (Thermo Fisher) and 200 µg of the purified rabbit IgG was labeled with peroxidase using a Peroxidase Labeling Kit (Dojindo Laboratories) and dissolved in 200 µL of storage buffer according to the manufacturer’s instructions. Polyclonal antibodies against N-terminal salusin-β were raised in chickens by immunization with a synthetic N-terminal salusin-β sequence, AIFIFI-[Cys^0^]^2^, and were extracted from egg yolk using a Thermo Scientific Pierce Chicken IgY Purification Kit (Thermo Fisher).

### Salusin-β-depleted human plasma

Plasma samples obtained from healthy subjects were circulated within a 60-cm polypropylene tube (outer diameter 4 mm, inner diameter 2 mm) using a peristaltic pump for 2 h at room temperature (RT). This allows free salusin-β to be removed by adherence to the inner surface of the tube. The plasma was then centrifuged at 1500 × *g* for 10 min and the supernatant stored at −30 °C until required for ELISA. Depletion of endogenous salusin-β was confirmed by the following two criteria: 1) failure to detect the native full-length salusin-β sequence using mass spectrometry and a non-tryptic native peptide identification approach; and 2) failure to detect salusin-β-like immunoreactivity using the specific antibodies described above.

### Sandwich ELISA to directly measure plasma free salusin-β

Flat-bottomed 96-well black microtiter plates (Porvair Sciences) were coated with 100 µL of 0.1 µg/mL anti-N-terminal salusin-β IgY as a capture antibody and incubated overnight at 4 °C. Plates were washed three times with PBS containing 0.05% Tween 20 (PBS-T, pH 7.4) and then blocked with 5% (w/v) skim milk in Tris-buffered saline for 2 h at RT. Salusin-β (Peptide Institute) was reconstituted in PBS containing 0.025% NP-40 to avoid its adherence to experimental plastic tubes and tips, and serially diluted to between 1 and 10 nM. Ten-microliter plasma samples were diluted with 40 µL of 0.025% NP-40/PBS, while 10 µL of salusin-β-depleted plasma samples were added to 40 µL of standard salusin-β solution serially diluted with 0.025% NP-40/PBS. This was added to 50 µL of peroxidase conjugated anti-C-terminal salusin-β IgG prediluted to 1:500 with 0.025% NP-40/PBS and incubated for 30 min at 37 °C. Microtiter plates precoated with anti-N-terminal salusin-β IgY were then washed with PBS-T three times, 100 µL of immune complex mixtures were applied to the wells, and the plates further incubated for 1 h at RT. The plates were then washed with PBS-T four times, 120 µL of 0.5% 3-(4-hydroxyphenyl) propionic acid containing 0.005% H_2_O_2_ added, and the plates incubated again for 1 h at RT. The reaction was then stopped by adding 0.1 M glycine-NaOH solution (pH 10.3) and the fluorescence measured at an excitation wavelength of 320 nm and an emission wavelength 405 nm on a SpectraMax M2 microplate reader (Molecular Devices).

### Clinical protocols

Stored plasma samples obtained using clinical protocols performed previously^2, 3^ were used for measuring the free salusin-β levels. These include: 1, postural changes (4 men and 2 women); 2, circadian variation (18 men and 9 women); 3, oral water loading (3 men and 3 women); 4, intravenous hypertonic saline loading (3 men and 2 women); and 5, Valsalva maneuvers (9 men, 2 women). Seven healthy subjects (3 men, 4 women) performed the Valsalva maneuver on a different day exactly as described^3^, except that blood samples were collected at 0, 15, 30, 45 and 60 s. Oral glucose tolerance tests were performed in 10 subjects (4 men, 6 women) whose laboratory data revealed that they were non-diabetic. After a 10-h overnight fast, all of the subjects ingested a solution containing 75 g of glucose and venous blood was drawn at 0, 30, 60, 90, 120, and 180 min.

Diabetic patients received routine systemic evaluation, including serum biochemical analysis^40, 41^. Patients were also evaluated for their glycemic excursion profiles using continuous glucose monitoring (CGM; CGMS® System Gold or Minimed iPro2® CGM, Medtronic Minimed)^33, 44^. All 576 glucose values obtained at 5-min intervals during 48-h CGM recordings were used to calculate the mean glucose or the glycemic SD, and this was compared against the plasma free salusin-β level for each patient.

### Statistical analysis

Data are expressed as the mean ± SE. Differences within groups or over time courses were examined for statistical significance using a one way ANOVA and *post hoc* comparisons were analyzed using a Mann-Whitney U test or a Wilcoxon signed-rank test. These analysis were performed using GraphPad Prism 5 (GraphPad Software). Multivariate analyses were performed using JMP ver. 5.0.1a (SAS, Cary, NC, USA) employing age, gender, diabetes, panhypopituitarism and body mass index as an explanatory variable and plasma free salusin-β level as an objective variable. Test results with *P* < 0.05 were considered as statistically significant.
